# Apple Waste/By-Products and Microbial Resources to Promote the Design of Added-Value Foods: A Review

**DOI:** 10.3390/foods14111850

**Published:** 2025-05-22

**Authors:** Hiba Selmi, Ester Presutto, Martina Totaro, Giuseppe Spano, Vittorio Capozzi, Mariagiovanna Fragasso

**Affiliations:** 1 Department of Agriculture Food Natural Science Engineering (DAFNE), University of Foggia, 71122 Foggia, Italy; ester.presutto@unifg.it (E.P.); martina.totaro@unifg.it (M.T.); giuseppe.spano@unifg.it (G.S.); 2 Institute of Sciences of Food Production, National Research Council (CNR) of Italy, c/o CS-DAT, Via Michele Protano, 71122 Foggia, Italy; vittorio.capozzi@cnr.it

**Keywords:** fruit, pomace, bioactive compounds, bioprocessing, fermentation, lactic acid bacteria, lactobacilli, yeast, non-*Saccharomyces*, circular economy

## Abstract

Apple fruit is among the most consumed fruits in the world, both in fresh and processed forms (e.g., ready-to-eat fresh slices, juice, jam, cider, and dried slices). During apple consumption/processing, a significant amount of apple residue is discarded. These residues can also be interesting materials to exploit, particularly for direct valorization in the design of added-value foods. In fact, apple waste/by-products are rich in essential components, including sugars, proteins, dietary fibers, and phenolic compounds, as they comprise apple peels, seeds, and pulp (solid residue of juice production). In this sense, the current review paper presents an overview of the nutritional composition of apple waste/by-products, and mainly apple pomace, highlighting their application in producing value-added products through microbial biotechnology. If appropriately managed, apple by-products can generate a variety of useful compounds required in food (as well as in feed, pharmaceutics, and bioenergy). Recent strategies for the synergic use of apple waste/by-products and microbial resources such as lactic acid bacteria and yeasts are discussed. This review contributes to defining a reference framework for valorizing apple waste/by-products from a circular economy perspective through the application of bioprocesses (e.g., fermentation), mainly oriented towards designing foods with improved quality attributes.

## 1. Introduction

Apple fruit represents the third most important fruit produced around the world, behind only bananas and watermelons. According to USDA data, in the 2023–2024 crop year, apple production accounted for about 84 million metric tons, with China being the primary producer, contributing about 57% of global production, followed by the European Union, contributing 13% of global production [1]. This fruit is cultivated worldwide in temperate, subtropical, and tropical environments [2]. Therefore, thousands of apple cultivars are grown to produce an ample variety of apples for the fresh market and a broad range of agro-food products [3].

**Figure 1:**
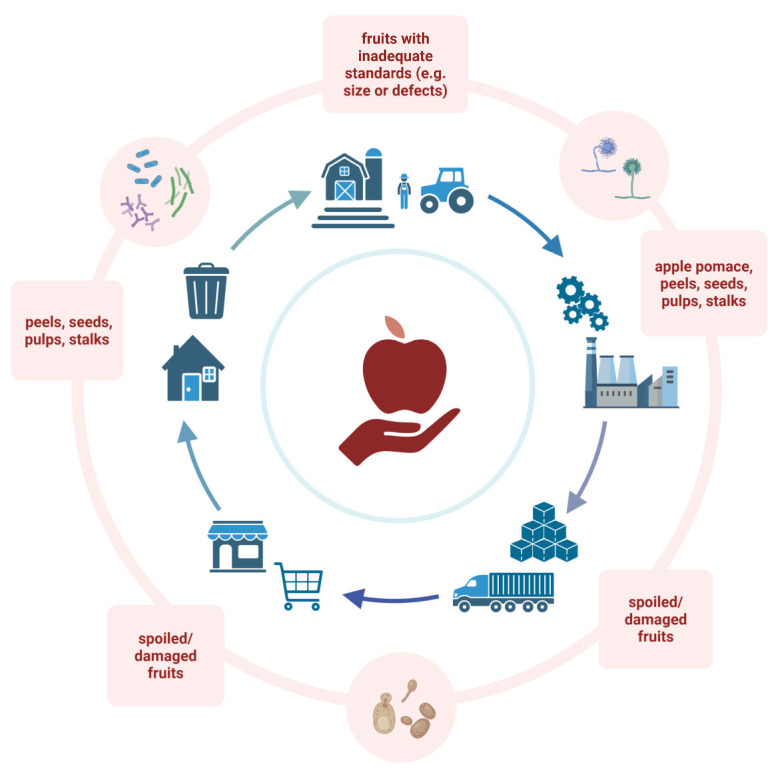
Schematic representation of the lifecycle of apples through primary production, processing, distributing, retail, and final use. The main by-products of each step are reported. It is important to highlight the advantages of microbial bioprocessing in the valorization of apple residues. Created in BioRender. Capozzi, V. (2025) https://BioRender.com/16uemp0.

Apples can be commercialized in both fresh and processed forms. Bruised, damaged, and defective apples are first discarded, and during apple manufacturing, peels, seeds, stems, and pulp are further rejected as by-products [4]. Apple by-products are the residual materials generated during apple processing, which might be considered as waste products, along with unused, defective, and discarded fruits, if not managed properly [5]. The use of preservatives, manufacturing and cooking processes, including pasteurization, and the addition of additives can reduce the spoilage of fresh fruits [6]. Nevertheless, a significant quantity of apples is discarded along the food supply chain, including the harvesting and processing stages [7]. Currently, the greater part of apple waste/by-products is used to feed animals [8] and as an agricultural fertilizer [9]. Despite the positive adaptation of the apple industry to the circular economy model, there is a lack of eco-friendly route implementation for several manufacturing outputs, which are different from the final products. In fact, apple consumption/processing generates a large number of valuable products that need to be recovered [10], considering the generic production lifecycle of apples for human production, starting from primary production (i.e., fresh products), followed by apple processing (e.g., ready-to-eat fresh slices, juice, jam, cider, and dried slices), distribution/logistics, and then consumption (Figure 1). Corresponding to the main production phases, the main waste products and by-products that are characteristic of each manufacturing step are reported in Figure 1. This schematic map is helpful in highlighting the production phases in which the main by-products and waste products of apple are generated, underlining that the aim of this review is to present evidence from the scientific literature for how microbial resources can contribute to valorizing these matrices, particularly for potential re-use in food production. By schematizing the apple production outputs potentially biotransformed into inputs for other products through the application of selected microbes, the figure below highlights the advantages of microbial bioprocessing in the valorization of apple residues, offering a perspective consistent with the green transition of apple production systems.

To provide information on the relevance of these resources, for example, apple pomace, the remaining solid matter after apple processing accounts for approximately 25% and 30% of the entire apple weight [11]. Additionally, this by-product contains various bioactive components that have received attention due to their potential to reduce inflammatory reactions and control cancer development [12]. Therefore, the extraction/valorization of these compounds is essential, and recent studies have focused on green extraction methods oriented towards low energy consumption and the use of environmentally friendly chemicals [7,13]. In this regard, novel technologies have emerged for re-using apple waste/by-products which are oriented towards specific chemical targets, including chemical and physical treatments such as enzyme-assisted extraction, ultrasound-assisted extraction, microwave-assisted extraction, and pulsed electric field extraction [14,15,16]. These methods can be utilized in combination in order to increase the availability of the compounds of interest [17]. For example, enzyme-assisted extractions can be employed as general pre-extraction or extraction methods to improve efficiency; these processes are based on using hydrolytic enzymes to hydrolyze cell walls or components [18]. In grape pomaces, cellulase, pectinase, and hemicellulose have shown extraction selectivity and efficiency toward their targets, including catechins, anthocyanins, and phenolic acids [19]. On the other hand, microwave-assisted extraction is known as a fast method. This technique can be utilized in closed or open systems; it is based on energy transfer via molecular interactions leading to moisture evaporation by heat [20]. On the contrary, ultrasound-assisted extraction is based on the physical disruption of plant cells using ultrasonic waves and the release of bioactive compounds in the solution [16]. In order to maximize phytochemical yield extraction from fruit peels, ultrasound-assisted extraction is widely applied in combination with other extraction techniques [21,22]. Pulsed electric field-assisted extraction is a non-thermal method and requires short- and high-voltage electric pulses, resulting in cell disruption [23]. However, these techniques require high equipment costs, are unsuitable for all samples, risk degrading volatile compounds, and are difficult to scale [24,25]. The purpose of scalability is to ensure that when demand grows, the process can quickly adapt to meet new requirements while retaining optimal efficiency, performance, and resource use [26]. Nevertheless, some green extraction technologies require a pretreatment step. Thus, reagents/chemicals, long pretreatment times, and particular optimization conditions are still needed. In addition, the output of the technology strictly depends on the composition and the physicochemical properties of the biomass [27]. Therefore, in consideration of the limitations of chemical and physical treatments, and considering the importance of exploring food-grade re-use of this apple biomass, fruit waste/by-product microbial bioprocessing (i.e., fermentation) appears to be a promising and sustainable strategy for obtaining the desired products with high-value-added compounds that benefit the productive chain [28]. Microorganisms can convert fermentable sugars into chemicals, organic acids, enzymes, and other bioactive compounds of interest, especially for food application and cosmetic and pharmaceutical industries [29]. The choice of microbial strain, media composition, and the parameters associated with the process condition highly influence the feasibility of fermentation to this end. Several attempts have been made using various bacteria, yeasts, and filamentous fungi, exploiting, for example, the metabolic activity of strains belonging to the genera *Lacticaseibacillus, Lactiplantibacillus, Bacillus*, *Streptococcus*, *Saccharomyces*, and *Aspergillus* [30,31,32,33].

Microbial diversity in the development of nature-inspired solutions to the problem of valorizing fruit-associated matrices represents a supply of crucial resources, underlining the importance of appropriate isolation, characterization, and conservation actions in specialized collections [34,35,36]. Lactic acid bacteria represent fundamental bioresources on account of their antimicrobial properties, beneficial for biocontrol, and their ability to positively modulate the main aspects of overall food quality, including nutritional and functional attributes [37,38,39,40]. The other large heterogeneous category of microorganisms relevant to fruit biotransformations are yeasts, namely, *Saccharomyces* and non-*Saccharomyces* [41,42,43]. Gulsunoglu and her colleagues revealed that apple peel’s fermentation with *Aspergillus* spp. for 7 days enhances the phenolic and flavonoid contents, thus improving apple peel’s antioxidant activity, reaching 2780 mg TE in 100 g of dry matter [44]. Moreover, another study investigated our capacity to improve the nutritional value of apple pomaces with γ-linolenic acid and carotenoids through solid-state fermentation via two *Zygomycetes* fungal strains for 12 days. The results showed a simultaneous enrichment of apple pomaces with γ-linolenic acid and carotenoids. Solid-state fermentation using *Umbelopsis isabellina* exhibited a 12.7%-higher γ-linolenic acid yield production compared to *A. elegans*, while the strain *A. elegans* showed higher carotenoids and phenolic antioxidants productivity than *U. isabellina* [45]. Likewise, Paniagua-García and others succeeded in producing lactic acid from apple pomace using thermotolerant bacteria, namely, *Heyndrickxia coagulans* and *Geobacillus stearothermophilus* strains, achieving a yield of 0.86–1.01 g/g [46]. Additionally, producing propionic acids becomes possible through the use of apple pomace as a substrate for *Propionibacterium freudenreichii* strains [47]. Pomace derived from apple juice was assessed as a suitable compound with which to design a novel formulation of microbial media growth [48]. In this regard, the co-culture of *Bacillus subtilis* and *Bacillus pumilus* was enhanced on apple pomace media, and the optimal pectinase activity doubled (11.25 IU/mL) [49]. Lactic acid bacteria strains were utilized as well; commercial starter lactic bacteria cultures were cultivated in apple pomace media or media supplemented with apple pomaces, and the results demonstrated apple pomace’s ability to generate a variety of compounds, such as phenolic compounds, enzymes, and organic acids [50,51]. Furthermore, the single-cell protein technique has emerged as an interesting method with which to valorize apple pomace; it refers to dead microbial cells or total protein extracted from the pure microbial culture of selected microbes, such as bacteria, algae, and filamentous fungi [52]. Strains belonging to *Aspergillus niger*, *Candida utilis*, *Geotrichum candidum*, *Bacillus subtilis*, and lactic acid bacteria showed strong a symbiotic effect in the apple pomace matrix, and all strains were capable of increasing the pure protein content after fermentation [53]. Yeasts including *Saccharomyces cerevisiae* were also capable of increasing the essential amino acids yield in fruit wastes [54]. Therefore, single-cell proteins are commonly used in animal and human feeds as dietary supplements [55,56,57].

The present review paper aims to provide an overview of apple waste/by-products’ possible valorization, with particular reference to food-grade bioprocessing solutions oriented towards the use of microorganisms (e.g., bacteria, yeasts, and filamentous fungi) as transformation agents. This review presents an integrated treatment, highlighting, organizing, and summarizing information/knowledge on (i) the composition of the main apple by-products; (ii) the use of these matrices as a source of biomolecules of interest; (iii) microbial diversity in the generation of novel foods from apple by-products; and (iv) the study of apple by-products with the aim of supporting the growth of desired microbes. The contribution concludes with (v) an overview of several case studies on the synergistic use of apple by-products and microbes to promote the design of value-added foods, highlighting various opportunities, and (vi) limitations associated with this type of valorization pathway.

## 2. Apple By-Products Composition

Apple appears to be one of the most beneficial foods in terms of its balanced nutritional composition, and it is known to have bioactive metabolites with oxidants and health benefits, including polyphenols [58]. Many apple cultivars are listed in the European apple inventory, thus reflecting a broad range of variability in fruit quality [59]. It is well known that the nutrient content of each crop varies depending on the cultivar species, age, soil quality, and climatic variations [60]. Indeed, the fruit’s appearance is the first purchase-driving trait that influences consumers’ consumption decisions. In other words, consumers assess apples first by their appearance (color, size, shape, absence of defects) and then by their eating quality, known as their “internal quality”, which includes texture and firmness, acidity, starch content, soluble solids, and nutritional value (Table 1) [61].

Water is the main component in all apple varieties, with a content of about 81% of the total fresh form. Moreover, apple fruit provides a moderate calorie content (55 kcal/100 g) [77], though proteins and lipids did not significantly contribute to the energy provided. Sugars are found in considerable concentration (about 11.8 g in 100 g fresh apple) [77], in which fructose and glucose are dominant when compared to sucrose and lactose [83]. Dietary fibers are also available in apples in both soluble and insoluble forms [84]. It is noteworthy that consuming apples with peels is preferable as the dietary fiber content of apple peel is twice to three times that of apple flesh.

Nevertheless, fresh apples can easily deteriorate because of their short shelf life and the occurrence of spoilage pathogens. Typical examples of processed products from apple fruits include juices, sauces, jams, apple pies, apple cider vinegar, wine, and apple snacks (chips) [85]. Among all processed apple products, apple juice is the most produced product (65%). During apple processing, a large number of by-products are generated. The major by-product released is called apple pomace, which is constituted of a mixture of peel, core, seed, calyx, stem, and the soft tissue that contains the most nutrients, including carbohydrates, proteins, dietary fibers, and minerals [72]. Before juice preparation, skins, stalks, and pips could be removed, which might affect the composition of the pomace. Generally, apple pomace extract contains 4.5 g of total sugars in 100 mL. Fructose is the most abundant, followed by glucose and saccharose, with a value of about 2.4 mg/100 mL, 1.9 mg/mL, and 0.2 mg/100 mL, respectively [86]. These findings are in line with the results previously published in a Polish study [87]. Based on the elemental analysis, Hobbi et al. found that carbohydrates are the main compounds in apple pomace, with a value of 71% (*w*/*w*), followed by volatile compounds (92% *w*/*w*) [88]. Indeed, during juice making, cell wall rupture results in the release of sugars and organic acids into the liquid part (the juice), which explains the higher content of these compounds in juice compared to pomace and pulp [89]. Furthermore, it was reported that apple peels are richer in total phenolic compounds and total flavonoids, which provide higher antioxidant activity than apple pulps [90]. On the other hand, apple seed is a good source of lipids, with an abundance of linoleic acid and oleic acid [91]. However, the concentration of fatty acids might vary in apple seeds according to cultivar. Minerals and vitamins are essential micronutrients for the function of the human body, and they are required in small amounts.

Minerals are commonly found in apple fruits, including potassium, magnesium, calcium, sodium, phosphorus, and trace elements of zinc, manganese, copper, and iron. It has been highlighted that apple pomace is rich in potassium (449.0 mg/100 g), phosphorus (50.0–950.0 mg/100 mL), and calcium (50.0–150.0 mg/100 mL) [92]. Remaining in the field of micronutrients, with regards to vitamin content, apple fruit is a rich source of B, A, C and E vitamins. Studies have revealed that vitamin C content is twice as high in apple peels than in the inner parts of the apple [93]. In apple peels, the ascorbic acid content ranges between 2.7 and 56.0 mg/100 g of fresh weight, while in pulps, the concentration was reduced to 0.1–13.9 mg/100 g of fresh weight [94]. Vitamin E is more available in apple seeds than in other parts of the apple [95]. Apple fruit contains a variety of molecules with health-promoting attributes: those belonging to polyphenols are predominant, and the total polyphenol concentration significantly varies according to the apple cultivar, the year of harvest, and the manufacturing process. For example, “Red delicious” apple peels (1187.0 mg/100 g) are four times richer than “Granny smith” apple peels (304.0 mg/100 g) in terms of total polyphenol concentration [96]. Indeed, it has been assessed that about 82% of the total polyphenol remained in the apple pomace after fruit processing [73]. Further study indicated that apple peels are an excellent source of phenolic acids compared to apple pulp. Chemical analysis revealed the availability of protocatechuic, vanillic, t-ferulic, and 3-(4-hydroxyphenyl) propionic acid in considerable amounts in the peel of all of the examined apple varieties [97].

## 3. Valorization of Apple By-Products as a Source of Highly Valuable Materials

The valorization of residues associated with apple production and their processing through solutions inspired by the bioconversions undertaken by microorganisms represents a family of interesting solutions in the promotion of the green transition of food systems. It is important to highlight that the chemical complexity/diversity varies with the apple residues. The apple pomace, for example, is a heterogeneous mixture of peels, cores, seeds, and stems, and it contains a high content of water and insoluble carbohydrates, mainly cellulose, hemicellulose, and lignin. Simple sugars, including glucose, fructose, sucrose, and small amounts of minerals, proteins, and vitamins, are all a part of the apple pomace composition [98]. It is also important to point out that this composition might vary based on the apple variety and the type of processing applied for juice extraction, especially how many times the fruits were pressed [99].

**Figure 2:**
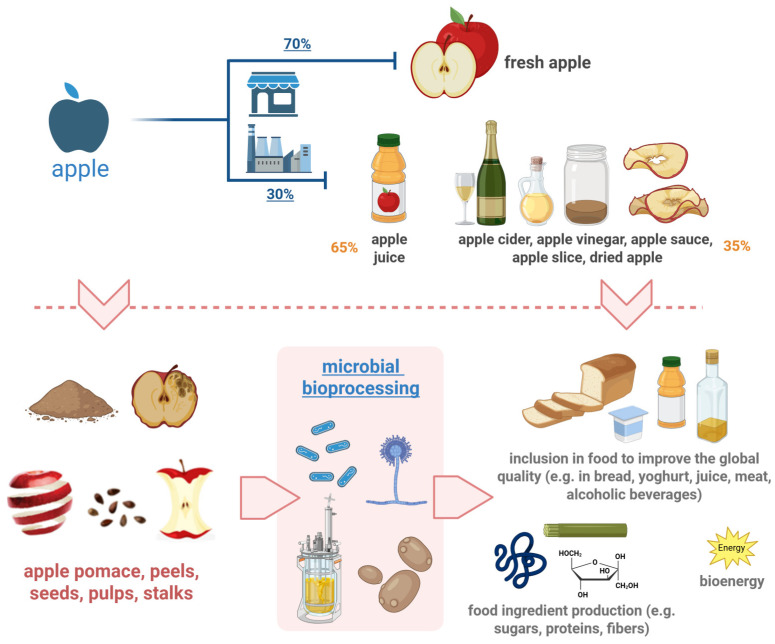
Overview of residues generated from apple processing, focusing on microbial bioprocessing strategies for biorefinery application. The apple industry can generate a number of apple by-products, including peels, seeds, pulps, and stalks. The introduction of microorganisms leads to the valorizing of these residues and thus the generation of high-value-added products, the creation of unconventional foods, and the development of bioenergy. Created in BioRender. Capozzi, V. (2025) https://BioRender.com/agxyd43.

In general, in valorizing a microbial-based solution, the apple waste by-product might be exploited for (i) recovering valuable compounds (e.g., for use as food ingredients of interest for new product development), (ii) developing new products that include high-value-added compounds, and (iii) for energy production (a subject that is beyond the scope of this review, for which we will provide only a brief overview considering the potential overlapping of different approaches) (Figure 2).

Numerous studies have reported that fruit pomaces are richer in bioactive substances than fruit juices [100]. Attempts have been made to generate several value-added products from this kind of matrix, comprising enzymes, single-cell proteins, aroma compounds, bioethanol, organic acids, biopolymers, and microbial biomass [101]. In this context, pectinase production in *B. subtilis* and *B. pumilus* co-culture was optimized by using apple pomaces as the only carbon source [49,102]. The suitability of apple pomace for cellulase production was estimated using solid-state fermentation models. Experiments showed that apple pomace possesses high initial cellulase content, reaching a maximum activity recovery of about 104 ± 27% after extraction with distilled water at a ratio of 1:2 [103]. Recently, several published works have reported the production of sustainable biomaterials from apple by-products [45,104,105]. As an example, pectin, one of the major components of dietary fibers that occur in apple pomace, is a soluble viscous fermentable fiber frequently employed as a gelling agent in the food industry [10]; it also serves as a biopolymer, preservative, anticorrosive agent, and protective agent for many different kinds of surface [106]. Teleky and his team have succeeded in generating a biodegradable film-forming solution enriched with organic or phenolic extracts from apple by-products [106]. Another study aimed to develop edible packaging films prepared from apple pectin and incorporated with apple and blackcurrant fragmented pomace. The results revealed that films with pomace powder were thicker, darker in color, and more mechanically tolerant compared to the control (film with only apple pectin) [107]. Likewise, the addition of apple pomaces to biodegradable films obtained from cassava starch enhanced their mechanical properties and demonstrated an ability to increase shelf life by exerting antioxidant and antimicrobial activities [108]. Further studies have focused on developing bio-based films and 3D objects from apple pomaces [109,110,111]. Researchers have managed to prepare 3D biomaterials from apple pomaces via the compression molding method, suggesting the suitability of this new material in edible packing and tableware [109].

Bioethanol is widely used as a solvent, fuel, and feedstock compound with numerous industrial applications. Apple pomaces can be an interesting alternative with which to obtain second-generation bioethanol and to minimize their environmental impacts [112]. With high amounts of carbohydrate content, it has great potential in producing bioethanol through anaerobic microbial fermentation. In this sense, Pathania et al. succeeded in producing bioethanol from treated apple pomace after co-culturing *Saccharomyces cerevisiae* and *Scheffersomyces stipitis* yeast cells, with a bioethanol yield of 44.56 g/L [113]. Likewise, it was possible to produce ethanol via *Pichia stipites* after the enzymatic treatment of apple hemicellulose. The maximum bioethanol concentration was found in the 10% (*w*/*v*) initial apple pomace (14.3 g/L) [114]. Recently, ethanol was recovered using yeast *Saccharomyces cerevisiae* UCLM S 377 from apple pomace; the maximum ethanol concentration was 31.30 g/L, with an initial sugar content of about 108.07 g/L [115]. Moreover, it has been reported that adding inexpensive soluble soy protein to apple pomace can significantly increase the enzymatic hydrolysis of complex apple carbohydrates and, consequently, economical bioethanol production [116].

Additionally, apple pomaces have been assessed for biogas production after the biorefinery approach. In other words, the obtained residue from apple pomace anaerobic digestion was mixed with residues from biobutanol fermentation, and the obtained mixture was utilized for biogas production. Consequently, the concentration of bioethanol from different bacterial and yeast strains was about 50 g/L, while the methane obtained from residues of bioethanol and biobutanol production was approximately 463 mL/g of the volatile solids added and 290 mL/g of the volatile solids added, respectively [117]. Furthermore, the anaerobic digestion of apple pomace in semi-continuous mode using a microbial community composed of bacteria (97.5%) and *Archaea* (2.5%) produced 36.61 L of methane from 1 kg of the total removed volatile solids. This methane yield can generate 1.92 kWh/ton of electricity and 8.63 MJ/ton of heat, avoiding 0.62 kg CO_2_ eq/ton apple pomace submitted to anaerobic digestion [118]. A recent study published by Abbasi-Riyakhuni and his colleagues proposed two valorization scenarios in order to enhance biomethane yield. Both methods included the integration of hydrothermal and organic acid pretreatment as the first step. The first strategy proposed the anaerobic digestion of liquors and solid residues, while the second strategy included the fungal cultivation and anaerobic digestion of both liquors and solid residues. As outputs, anaerobic digestion reduced 1.1 ton CO_2_ eq in greenhouse gases emissions and saved 259 USD associated with the social cost of carbon, with a production of 344.9 m^3^ of biomethane (89.1 L gasoline equivalent); while the combination of anaerobic digestion with fungal cultivation reduced 2.9 ton CO_2_ eq in greenhouse gases emissions and saved 811 USD associated with the social cost of carbon, generating 36.1 kg of fungal biomass, 36.1 kg of mycoprotein, and 178.1 m^3^ of biomethane from 1 ton of apple pomace [119].

Several bioproducts can be generated, which can diversify industry revenues and reduce waste volume [120]. Also, in this case, the choice of microorganism, the fermentation type, the composition of the apple substrate, and the applied processing parameters are key factors in shaping the process efficiency and the desired final product’s properties. An effective pretreatment can sometimes be needed to fully introduce apple residues into the biorefinery concept. However, pretreatment methods can generate toxic compounds that limit fermentation and reduce efficiency [121]. Several challenges are associated with translation from laboratory-scale to large-scale commercial production. Extensive and more in-depth technoeconomic studies are needed in order to design and develop industrial processes to support the transition to the circular economy, aiming to reduce economic costs and environmental pollution.

Various studies worldwide have targeted the usage of apple pomaces to enhance functional ingredients as a means of nutrition enhancement. Phenolic and terpenic contents have been assessed in pomaces of four different apple cultivars using HPLC coupled to different detection modes, including UV, ELSD, or MS. The analysis revealed the richness of all apple pomace extracts in bioactive compounds that exert antioxidant activity [122]. Similarly, industrial apple pomace extracts’ anti-inflammatory and anticancer effects have been evaluated, proving their potential to be applied as a functional ingredient [123]. In this context, Sobczak et al. utilized apple pomace as a valuable raw material in food production. Their study included a description of the recipe for the production of four food products based on apple pomace and wheat bran [87]. Indeed, incorporating apple pomace in baked products is thought to boost both dietary fiber content and health benefits [124,125]. It has been reported that *Pediococcus acidilactici* LUHS29 cells, immobilized in apple pomaces, support the bacterial cells’ stability, fortify the barley with phenolic compounds during fermentation, and reduce 10% of the acrylamide content in bread [126]. Another study revealed an improvement in the total polyphenols content and antioxidant activity in wheat bread supplemented with 10% apple pomace powder, though the administration of pomace did not affect the sensory properties of the final product [127]. Contrariwise, it was observed that cookies prepared with apple by-products showed satisfactory results in terms of their sensory properties and the availability of bioactive molecules, especially phenolic compounds [128].

Along with apple by-products, incorporating apple pomace into dairy products increases their nutritional content and positively impacts their texture and sensory traits. Further studies were interested in developing yoghurts with an improved structure, texture, and antioxidant potential using apple pomaces obtained from juice manufacturing [129]. The physicochemical and sensory analysis affirmed a significant increase in antioxidant activity and an improvement in the yoghurt’s textural properties during storage [129]. Likewise, the addition of apple pomace favored aggregation of casein micelles at an early stage of milk fermentation, the yoghurt texture was reinforced, and after 28 days of storage, a significant increase in gel firmness and cohesiveness was observed when increasing the apple pomace concentration from 0% to 1% [130]. Regarding meats, apple pomace can be used as a fortifying agent and a color enhancer for beef burgers. At concentrations of 4% to 8%, color and sensory analysis of the fortified products were graded better than the control (0% of apple pomace), and dietary fiber and phenol contents were improved [131]. A recent Polish study suggested the utilization of freeze-dried apple pomace as a component in meat, thus reducing the redness during refrigerated storage of baked meat and inhibiting lipid oxidation after the 15th day of storage [132]. Whereas, in turkey sausages, the incorporation of freeze-dried apple pomace at a concentration of 3% resulted in a significant decrease in moisture and protein content and an increase in total phenolic content and fibers [133]. Additionally, fortifying Italian salami with 7% or 14% dried apple pomace was found to increase its nutritional properties, with low fat and a low caloric value [134].

## 4. Microbial Diversity in the Generation of New Foods from Apple By-Products

### 4.1. Apple Juices

The fermentation of apple juices can be a promising alternative with which to generate high-value-added products. In this regard, apple juice was explored as a growth medium to cultivate the probiotic *Lactiplantibacillus plantarum* PCS 26 [135], and it was found that apple juice fermented by lactic bacteria strains increased the quantity of antioxidants and improved the content of bioactive compounds [136]. Both *Lacticaseibacillus casei* and *Lactobacillus acidophilus* were utilized to develop and optimize fermented apple juices. The obtained probiotic beverage was characterized by a caramel color, apple aroma, and acidic apple taste, with a prolonged storage period reaching 28 days [137]. Indeed, the aromatic profile of fermented juices with *L. acidophilus* ATCC 4356 was characterized by the presence of more than 53 volatile compounds, which positively affected the sensory profile of the final product [138]. Recently, three probiotic lactic bacteria strains belonging to *Lpb. plantarum*, *L. acidophilus*, and *Lcb. casei* species were selected to ferment apple juices. All strains grew well, and after 72 h of lactic fermentation, there was a change in the apple juice color and the browning index value increased and then decreased rapidly, which was consistent with the changes in organic acids and the sugar profile. Indeed, the ultrasonication of *Lactiplantibacillus plantarum* strains during apple juice fermentation can regulate the derivations of phenolics and organic acids content [139] and improve the antioxidant and antimicrobial capacities of the juice by metabolizing phenolic compounds and producing lactic acid [140]. Likewise, Lorenzini and her team investigated the capacity of yeast strains to ferment apple juice and produce volatile compounds. The findings revealed that yeast could be used in single or in mixed culture as a co-starter for cider making, and the amount of fatty acids and acetate esters was much higher in cider produced via *Saccharomyces* strains than in cider obtained via non-*Saccharomyces* yeasts [141]. In line with these findings, Kanwar and Keshani observed a variation in the ATF1 gene, which is responsible for ester synthesis during fermentation, in *Saccharomyces cerevisiae* strains, which could be a pivotal factor for aroma and flavor diversity [142]. Apple juice fermentation using *Saccharomyces cerevisiae* can generate ethanol in the final product, which is the main carcinogen in alcoholic beverages. Nevertheless, the de-alcoholization process might retain functional substances, including vitamins, minerals, and antioxidants. On this subject, Huang et al. analyzed the fermentation of apple juice with *Saccharomyces cerevisiae*, and after de-alcoholization, *Lactiplantibacillus plantarum* strains were added to enrich the final product. After LAB fermentation, the content of riboflavin, pantothenic acid, vitamin B6, and vitamin C were increased three times, reaching 68.69 mg/100 mL, 4.88 mg/100 mL, 1.17 mg/100 mL, and 1.97 mg/100 mL, respectively [143].

### 4.2. Apple Pomaces

Apple pomace is rich in dietary fibers with a high bioaccessibility of phenolic compounds [144], making pomaces an excellent substrate for bioprocesses, being rich in sugars which might serve as carbon sources for various microbes [10,99]. Bacteria, yeast, and fungi have been cultivated for metabolite synthesis, implicated in food and textile processing, the degumming of plant rough fibers, and the treatment of wastewater [99]. Nevertheless, apple pomace is very submissive/sensitive to microbial spoilage because of its high water content; it is also very susceptible to the rapid depolymerization and de-esterification of pectins. To stop and reduce this deterioration, it is necessary to dry the apple pomace immediately upon its production [73].

The fermentation of apple pomaces with complex probiotics of *Lactiplantibacillus plantarum* DPH, *Saccharomyces cerevisiae* SC9, and *Bacillus subtilis* C9 for six or nine days enhanced their antioxidant properties and significantly increased their beneficial metabolites, including amino acids, organic acids, gamma-aminobutyric acid, and serotonin [145]. Researchers found that lactobacilli with cell-membrane-associated β-glucosidase activity were able to transform apple pomace through fermentation and increase polyphenols and antioxidant availability [146]. A study assessed the ability of apple pomace to fortify wheat breads. It was found that the addition of 5% fermented apple by-products enhanced dough water absorption and stability, increasing dietary fiber content [147]. Another study revealed that apple pomace incorporation to about 20% increases the level of phenolic compounds and flavonoid content in baked products [148]. Indeed, Gumul and his team have incorporated apple pomaces in wheat bread at various concentrations (5, 10, and 15%) and have evaluated the chemical composition, physical characteristics, and sensory attributes of the final products. Results revealed a significant increase in bioactive compound content, including high antioxidant potential in gluten-free bread, after adding apple pomace (2.5–20 times) compared to the control [149]. Likewise, a study conducted by Alongi proposed the possibility of decreasing glycemic levels in biscuit products by substituting wheat flour with 10% or 20% (*w*/*w*) of apple pomaces. The unconventional biscuit presented a lower glycemic index of 65 and 60 after adding 10% and/or 20% of apple pomaces, respectively, compared to the control (classic biscuit, without apple pomace) [150]. Furthermore, apple pomace extracts can be added to baked products as a source of highly valuable molecules, allowing for the fabrication of functional foods with strong anti-inflammatory and antithrombotic properties [151]. The prebiotic potential of freeze-dried apple pomaces during soy milk fermentation has been investigated, and results suggest that this addition improves the polyphenolic content, antioxidant activity, and viable cell counts of the probiotic *Loigolactobacillus bifermentans* MIUG BL 16 [48]. Commercial *Lpb. plantarum* starters increased the content of phenolic compounds and antioxidants during apple pomace fermentation [50]. A recent study investigated the potential of lactic bacteria strains to transform apple pomace into an aromatizer for alcoholic beverages. Aromatic compounds were more concentrated in beer flavored with fermented apple pomace *Lacticaseibacillus rhamnosus* strains than in the control or in beer flavored with apple pomace [152]. Likewise, *Lactobacillus bulgaricus* and *Streptococcus thermophilus* strains have been utilized to produce Greek yoghurt enhanced with apple pomace syrup. The fortification of yoghurt with 1.25% apple pomace syrup increased the total polyphenols and antioxidant activities and was shown to be the most acceptable after sensory evaluation [153].

### 4.3. Apple Peels

Singh and his colleagues investigated the utilization of apple peels as feedstock for the production of alpha-amylase by the strain *Bacillus subtilis* BS1934 [154]. Likewise, Amorim et al. aimed to evaluate the suitability of apple by-products as carbon sources for bacterial cellulose production by a microbial consortium. It was found that the increase in apple waste content in the media (10% *w*/*v*) significantly increased the production yield [155]. Apple peels can be an interesting source of various valuable products; a study investigated the possibility of producing alginate from apple peels using *Azotobacter vinelandii* through solid-state fermentation [156]. Indeed, the fermentation of apple peels with *Aspergillus oryzae* has increased free phenolic compounds and antioxidant levels, and the fermented apple peels showed a prebiotic potential toward gut microbiota [157]. *Saccharomyces cerevisiae* can ferment apple by-products and produce pectin, and the obtained pectin samples expressed significant antioxidant activities and can thus be used as emulsifiers and rheology modifiers in foods [158]. 2-phenylethanol, a value-added compound widely used in industry due to its rose-like odor and antibacterial properties, can be bio-produced by *Pichia kudriavzevii* using apple by-products [159]. In this regard, Yang and his colleagues selected *Aspergillus niger*, *Candida utilis*, *Geotrichum candidum, Bacillus subtilis*, and lactic acid bacteria as the most suitable strains for apple by-products’ bioconversion into a nutritive animal feed rich in microbial protein. The co-culture at a ratio of 1:1 for apple by-products has significantly increased the microbial protein yield [53]. Apple peels can be used as a natural adsorbent for metal removal, including copper and chromium from wastewater [160]. Indeed, recent research focused on heavy metal fixation via apple waste as an alternative adsorbent. Results showed that apple pomace modified by potassium permanganate and apple pomace modified by sodium hydroxide were able to remove approximately 95% of zinc, cadmium, lead, and copper from wastewater [161].

## 5. Apple By-Products to Support the Growth of Desired Microbes

**Figure 3:**
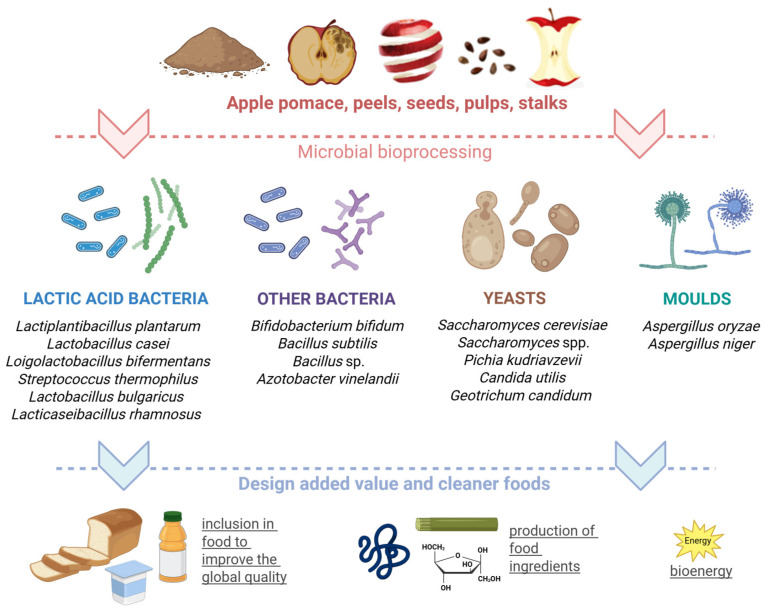
Overview of microorganisms involved in the bioprocessing of apple residues. A variety of microbes are able to grow and utilize apple by-products as substrates to produce high-value-added products. The choice of microorganism depends on the substrate composition and the desired final product. Created in BioRender. Capozzi, V. (2025) https://BioRender.com/fb59mk3.

In the last decade, apple by-products have emerged as a valuable reservoir of bioactive compounds that might enhance the growth of beneficial microbes, combined with their interesting ability to inhibit undesired Gram-positive and Gram-negative pathogenic bacteria [162]. Most developed applications involve batch fermentations, whereby the substrate and producing microorganisms are added to the system at time zero and are not removed until the process is complete [163]. A growing acknowledgement of the prebiotic potential of apple pomaces with respect to probiotic microorganisms has sparked a surge in in-depth studies (Figure 3).

In this sense, probiotics belonging to *Lactiplantibacillus plantarum*, *Lacticaseibacillus casei*, *Escherichia coli*, and *Saccharomyces* ssp. species were found to grow well in commercial culture media supplemented with apple pomace as the only carbon source, and after 48 h, the number of colonies reached about 10^8^ CFU/mL [164]. Further study revealed that propionic acid bacteria, i.e., the *Propionibacterium freudenreichii* T82 strain, can successfully grow in media consisting of waste materials such as apple pomace extract and potato wastewater. Findings affirmed that apple pomace and potato wastewater could serve as an affordable source of sugar and nitrogen, respectively [86]. At 2% apple pomace supplementation (*w*/*v*) in MRS broth, the prebiotic effect toward *Loigolactobacillus bifermentans* reached its maximum, i.e., about 4.7, after 21 days of storage at 4 °C [48]. Indeed, *Bifidobacterium bifidum* DSM 20,239 could utilize apple pomaces and release an important amount of short-chain fatty acids (SCFAs) (0.47 mmol/g) [165]. Another study, conducted in 2025, showed the ability of apple pomaces to stimulate the growth of the probiotic *Bacillus subtilis* and to increase their adherence ability [162]. Indeed, the prebiotic potential of apple pectin oligosaccharides was assessed using lactic bacteria strains; the results showed a strong adhesion capability after incubation with apple pomace hydrolysates, while they showed a significant reduction in the number of adhered pathogenic bacteria in a human cells model in the presence of apple pomaces [166]. After enzymatic treatment, oligosaccharides fraction with low molecular weight (MW < 30 kDa) showed higher prebiotic scores for both lactobacilli and bifidobacteria probiotic bacteria [167]. Likewise, the water-soluble fraction obtained from apple pomace after enzymatic hydrolysis showed a good prebiotic effect to support the probiotics *Lactobacillus acidophilus* DSM 20,079 and *Bifidobacterium animalis* DSM 20,105 [168]. Rößle et al. tested the ability of the probiotic *Lactobacillus rhamnosus* GG to survive in apple wedges and the effect of apple prebiotics, mainly oligofructose and inulin, on their survival rate [169].

It has been proposed that apple pomace administration can increase the diversity of microflora. For this purpose, Calvete-Torre and her colleagues fermented fecal samples collected from healthy donors and patients with Crohn’s disease in the presence of apple pomaces. Their results confirmed the capacity of apple pomace/pectin to promote significant species that are typically under-represented in patients with Crohn’s disease [170]. A 2022 study investigated the modulatory effect of pectin and apple pomace on human microbiota from healthy subjects and patients with inflammatory bowel disease (IBD) through fecal batch fermentation and 16S rRNA gene sequencing [163].

## 6. Case Studies on the Synergic Use of Apple By-Products and Microbes to Promote the Design of Added-Value Foods

Fermentation is an important and effective method for food processing and bioconversion. During fermentation, detrimental components within the substrate, such as anti-nutritional factors and toxins, can be reduced, and the nutritional/functional value can be effectively improved [171]. Several genera of microorganisms currently applied in food fermentation include lactic bacteria such as *Streptococcus*, *Lactobacillus*, *Lactiplantibacillus*, *Lactococcus*, *Leuconostoc*, and *Staphylococcus*; other bacteria, such as acetic bacteria, propionic bacteria, and *Bacillus*; yeasts like *Saccharomyces* and *Zygosaccharomyces*; and mold genera such as *Aspergillus* [172]. Generally, the fermentation leads to various end-products, including carbon dioxide, alcohols, and organic molecules. During alcoholic fermentation, yeasts hydrolyze hexose and disaccharides into ethanol and carbon dioxide in anaerobic conditions, generating small amounts of volatile compounds [173]. Indeed, *S. cerevisiae* cells are able to transform amino acids into novel volatile compounds through transamination and decarboxylation [174]. It is well established that lactic bacteria are among the most commonly employed microbes in the fermentation of different product categories, such as dairy products, vegetables, fruits, and meat. In the case of homofermentative lactic bacteria, sugar is converted into lactic acids to obtain energy. Meanwhile, heterofermentative lactic bacteria transform sugar into lactic acid, ethanol, acetic acid, and other organic acids [175]. Through acetic fermentation, *Acetobacter* spp. and *Bacillus subtilis* var. *natto* oxidize ethanol under aerobic conditions to generate acetic acids [176]. Moreover, propionic bacteria can utilize carbon substrate and produce propionic acid as the final product during fermentation [172]. Researchers expect apple residue to be introduced in numerous pharmaceutical, industrial, and agriculture fields, such as soil fortification [177]. Furthermore, the fermentation of apple by-products improves its value and generates high-value-added compounds (Table 2).

In this regard, incorporating apple fibers into yoghurt significantly increased *Lacticaseibacillus casei* ATCC 393 cells; in fact, these biomolecules acted as a protective matrix, preserving the viability of this probiotic strain [178]. Adding freeze-dried apple pomace powder to stirred-type yoghurt has improved the final products’ rheological properties and increased dietary fiber and phytochemical contents [179]. Likewise, incorporating apple pomace extracts into yoghurts has enhanced their antioxidant and anti-inflammatory profile [124]. Indeed, the heat treatment of apple by-products generates an aqueous extract with high polyphenolic content and antioxidant capacity.

A study analyzed the impact of adding freeze-dried apple pomace on the physicochemical properties and sensory features of meat products. It was observed that the meat products containing freeze-dried pomace had a higher saturated fatty acid content and higher lactic bacteria content, with an increase in the brightness of the baked meat [132]. In turkey sausage, freeze-dried apple pomace supplementation has been shown to significantly decrease pH, cooking loss, and yellowness and has improve the total phenolic content [133]. The use of apple by-products goes beyond alcoholic beverage manufacturing; it has been found that yeast fermentation leads to significant increases in the levels of volatile compounds and alcohols in alcoholic beverages [180]. After 15 days of fermentation, that was increase of 23% in total phenolic compounds without affecting the fermentation kinetics [181]. Moreover, Ricci et al. have exploited the potential of fermented apple pomaces as an aromatizer in the beer-making industry: after lactofermentation using *Lacticaseibacillus rhamnosus* and *Lacticaseibacillus casei* strains, the fermented pomaces were added as an innovative aromatizer [152].

Several findings proved the ability of apple peels to promote the proliferation of probiotic bacteria and to increase the availability of short-chain fatty acids (SCFAs) in human gut microbiota. The total SCFAs content in the apple peels group improved from 2.271 to 37.093 mM after 24 h [182]. Indeed, studies have revealed the antimicrobial potential of apple peels against Gram-positive and Gram-negative bacteria [183]. Dried apple peel powder administration in the DSS-induced colitis murine model succeeded in ameliorating the severity of DSS-induced colitis and showed preventive and curative effects on mitochondrial functions [184]. Likewise, apple pomace flour decreased body weight gain and blood glucose and improved glucose tolerance. Long-term supplementation with apple pomace flour (0.5% *w*/*w*) showed a significant improvement in glucose tolerance and a decrease in body weight (60% decrease in total weight) in mice exposed to a high-fat and sucrose diet [185].

Preclinical trials revealed health benefits related to consuming apple pomace as a functional ingredient in foods, including improved lipid metabolism, antioxidant, anti-inflammatory, and antiproliferative potentials [186]. An in vitro study revealed the therapeutic effects of apple pomaces, comprising their ability to reduce oxidative stress combined with their anticancer and anti-urolithic potential [187]. Indeed, apple peel extracts have shown cytotoxicity and antiproliferative potential, and within a concentration of 7.6 mg/mL, peel extracts significantly inhibit the proliferation of breast cancer cells [188]. Apple pomace extracts are likely to prevent and treat neurodegeneration diseases and ameliorate the expression of hypothalamus genes. Repeated treatment with apple pomace extract in mice for 7 days reversed the MK-801-induced impairment of associative and recognition memory and improved memory [189]. Numerous randomized controlled trials have investigated the clinical functions of apples and evaluated their effects on improving metabolic and cardiovascular markers compared to placebo or any alternative diet. It has been demonstrated that more than a week of apple and/or apple-derived products intake could reduce total cholesterol (TC), high-density lipoprotein (HDL), and low-density lipoprotein (LDL) levels when compared to a placebo-controlled diet [190]. Likewise, in vivo studies have evaluated the effects of apple peel extracts and apple fleshes on animal models. After mice were administered 250 mg/kg of apple extracts for 28 days, results showed lower blood pressure, improved endothelial function, and decreased insulin resistance compared with control mice [191]. Indeed, daily apple consumption allowed for a reduction in total and low-density lipoprotein cholesterol levels by 8.3% and 14.5%, respectively, and an increase in high-density lipoprotein cholesterol levels by 15.2% in the healthy group [192]. In a randomized, controlled, crossover trial, the consumption of two apples a day lowered serum cholesterol and improved cardiometabolic biomarkers in mildly hypercholesterolemic adults [193].

**Table 2:** Recent findings on apple by-products valorization through microbial bioprocessing.

Apple By-Product	Matrix Type	Species	Application	Reference
Apple fiber	Dairy products	*Lacticaseibacillus casei*	Incorporation into yoghurt Protective matrix for the probiotic strain	[178]
Freeze-dried apple pomace		Yoghurt starter culture	Improve the rheological properties Increase dietary fiber and phytochemical contents	[179]
Apple pomace extract		Yoghurt starter culture	Enhance antioxidant and anti-inflammatory potential	[124]
Freeze-dried apple pomace	Meat products	Autochthonous lactic bacteria	Increase in the brightness of baked meat	[132]
Freeze-dried apple pomace		Autochthonous Turkey sausage	Decrease pH, cooking loss, and yellowness Improve the total phenolic content	[133]
Apple pomace		Autochthonous Buffalo meat sausage	Improve physicochemical and sensory properties	[194]
Apple pomace powder		Autochthonous *Goshtaba* microbial resources (Traditional Indian meatballs)	Fat replacer: decreased fat content in the final product Improve sensory properties	[195]
Apple by-products	Fermented beverages	*Saccharomyces cerevisiae*, *Torulaspora delbrueckii*	Increase in the level of volatile compounds	[180]
Apple pomace		*Saccharomyces cerevisiae* r.f. *bayanus*	Fermentation kinetics not affected Higher alcoholic level Increase in polyphenol compound level	[181]
Apple pomace		*Lacticaseibacillus rhamnosus* and *Lacticaseibacillus casei*	Aromatizer in beer	[152]
Apple pomace	Baking products	*Weissella cibaria*, *Leuconostoc mesenteroides*, *Lactiplantibacillus plantarum*, *Saccharomyces cerevisiae*, *Hanseniaspora uvarum*	Increase in total and insoluble dietary fibers Extend the shelf life of the wheat bread	[147]
Apple pomace		Autochthonous microbes	Increase fiber content and antioxidant properties of extruded snacks and baked scones	[148]
Apple pomace			Production of gluten-free bread enriched with apple pomace Increase phenolic compound content	[149]

## 7. Limitations Associated with the Consumption of Apple By-Products

The consumption of added-value foods prepared with apple by-products provides nutritional/functional benefits, but there are several challenges to consider [72]. Most of the problems arise from the contaminations associated with the matrices of interest. For example, pesticide residues can be possible in apple peels. Indeed, to increase crop yields and prevent plant diseases and infection, pesticides are commonly applied directly on apple peels [196]. These chemicals can persist on the fruit surface or/and penetrate into the pulp. A recent study reported the availability of pirimicarb, captan, and cyprodinil in apple peels, with cumulative permeations of 90, 19 and 17 µg/cm^2^, respectively [197]. Moreover, it has been reported that pirimicarb showed a higher average transfer to the pulp than boscalid and deltamethrin, which remained on the apple skin [198]. Thermal treatment, washing, and cooking can control and reduce pesticide concentration [198,199]. Mycotoxins can be challenging while valorizing apple by-products, especially patulin, which can be found in dried apple by-products [200]. Recently, several attempts have been made to ensure the adsorption of heavy metals via apple pomaces. Studies have shown the ability of apple pomace to fix zinc, copper, lead, and cadmium with interesting yields [201]. Modified apple pomace can fix lead, zinc, copper, and cadmium with a maximum adsorption percentage of 99.7%, 99.3%, 99.2%, and 96.5%, respectively [161]. Indeed, the maximum absorption capacity of modified apple pomace was about 177 mg of lead per gram [202]. Apple seeds contain amygdalin, a naturally occurring cyanogenic glycoside, an aromatic cyanogenic compound that is non-toxic in itself, but when it is hydrolyzed, it can release cyanide, a highly toxic chemical [203]. The oral administration of amygdalin is related to developing severe adverse reactions, including vomiting, nausea, shortness of breath, and loss of consciousness. The mean lethal dose of amygdalin administered orally to rats was reported to be 0.88 g/kg body weight [204]. According to The World Health Organization, the acceptable daily oral dose of hydrogen cyanide (1 g of amygdalin released 59 mg of hydrogen cyanide) for adults (50–60 kg) is set to about 0.6–0.7 g [205]. It has been estimated that apple seeds contain 13.4–18.6 mg of amygdalin in each 100 g of dry matter, while apple pomaces have 0.1 mg of amygdalin in 100 g of dry matter [206]. Despite the high content of nutrients, apple by-products are rich in insoluble sugars (resistant starch) and dietary fibers that cannot be directly hydrolyzed. These molecules need to be pretreated and then hydrolyzed. Furthermore, the extraction and purification of select compounds will not be economically feasible with poor-quality products [4,207].

## 8. Conclusions and Future Perspectives

The transition to sustainable food systems represents one of the main challenges in the productive sector. For economic interest, diffusion, and scientific interest, apple production represents a particularly relevant field, as well as a study model. Apple processing waste/by-products—mainly pomace, peels, and seeds—represent an abundant and underutilized resource, rich in nutrients and biomolecules of interest, such as carbohydrates, phenolics, fibers, and micronutrients. Bio-solutions inspired by microbial-mediated conversions (fermentation by lactic acid bacteria, yeasts, and filamentous fungi) not only valorize these matrices but also enable the design of novel value-added foods and ingredients (e.g., fermented beverages, fiber-enriched dairy and bakery products, single-cell proteins, bioplastics, and bioactive extracts) with specific quality attributes. The integration of apple residues can help reduce waste, diversify industry revenues, and improve final product nutritional and sensorial profiles. It is crucial to underline the importance of microbial collections to improve the valorization of microbial diversity, guarantee experimental reproducibility, and promote biotechnological resources. Future work needs to focus on process optimization, technoeconomic assessments, and regulatory frameworks. In particular, it is important to monitor the safety aspects of production, ensuring a combination of economic and environmental sustainability, along with the social sustainability of the solutions developed. Finally, further exploration is required to upscale from lab-scale or pilot-scale experiments to industrial-level production, to unlock sustainable innovation in food systems, and to promote the circular economy.

## Figures and Tables

**Table 1:** The chemical composition of apple fruit and apple by-products according to recent published findings. Macronutrients and carbohydrates (simple and complex carbohydrates) were measured in grams per 100 g of fresh fruit or dried (as noted in the table) and are presented in percentages. Minerals were measured in mg per 100 g of fresh or dried material. n.d., not data; fm, fresh matter; dm, dry matter; GAE, gallic acid equivalent; *w*/*w*, weight/weight; Ref., references.

	Fat (g/100 g)	Protein (g/100 g)	Total Carbohydrate (g/100 g)	Total Fiber (g/100 g)	Insoluble Fiber (g/100 g)	Soluble Fiber (g/100 g)		Total Polyphenols (mg GAE/100 g)	Vitamin C (mg/100 g)	Ref.
Apple peel (dm)	2.7–14.8	2.8–4.5	60.0–81.9	40.3–43.9	11.9–25.5	14.7–32.0		1.1–9.6	60.3–63.4	[62,63,64,65]
Apple seed	19.7	35.3–40.1	n.d.	19.5–21.1	n.d.	n.d.		270.0–1744.0 defatted matter	nd	[66,67]
Apple pulp	10.0	4.7	79.0	3.0	n.d.	n.d.		22.0–62.0 fm	3.1–4.4 fm	[68,69,70]
Apple pomace	1.2–3.6	1.2–5.9	44.5–57.4 *	27.9–49.5	017.4–25.6	13.5–25.5		266.0–394.0 dm	12.0–52.0 dm	[63,71,72,73,74,75,76]
Apple pulp + peel	5.4	2.5	71.8	n.d.	n.d.	n.d.		10.8 dm	n.d.	[63]
Whole apple (fm)	0.2–0.3	0.1–0.2	10.0	2.0–2.1	n.d.	n.d.		n.d.	6.7	[77,78]
	**Sodium (mg/100 g)**	**Potassium (mg/100 g)**	**Calcium** **(mg/kg)**	**Phosphorus** **(mg/100 g)**	**Magnesium (mg/100 g)**	**Iron (mg/100 g)**	**Zinc (mg/100 g)**	**Copper (mg/100 g)**	**Manganese (mg/100 g)**	**Ref.**
Apple peel	<0.01–0.4	800.0–1100.0	1000.0	100.0	100.0	1.3–1.4	1.0	0.04–0.2	0.02–0.5	[65,79,80,81]
Apple seed	n.d.	650.0	270.0	72.0	51.0	11.0	4.4	0.2	0.5	[82]
Apple pulp	0.6–0.8	68.7	0.9–3.9	n.d.	0.6–1.8	n.d.	n.d.	n.d.	n.d.	[68]
Apple pomace	39.3	872.8–925.0	55.6–92.7	50.0–112.0	20.0–61.6	2.4–23.0	0.9–1.8	0.6–0.9	0.4–1.8	[73,75]
Whole apple	<1.0–1.0	59.0–95.0	2.0–5.0	6.0–9.0	3.0–4.7	<0.1–0.1	0.02	0.01–0.02	0.02–0.03	[77,78]

* Glucose: 2.5–22.7 g/100 g. Fructose: 11.5–49.8 g/100 g.

## Data Availability

No new data were created or analyzed in this study.
